# Cerebellar Direct Current Stimulation (ctDCS) in the Treatment of Huntington's Disease: A Pilot Study and a Short Review of the Literature

**DOI:** 10.3389/fneur.2020.614717

**Published:** 2020-12-03

**Authors:** Tommaso Bocci, Davide Baloscio, Roberta Ferrucci, Ferdinando Sartucci, Alberto Priori

**Affiliations:** ^1^“Aldo Ravelli” Center for Neurotechnology and Experimental Brain Therapeutics, Department of Health Sciences, University of Milan & Azienda Socio-Sanitaria Territoriale Santi Paolo e Carlo, Milan, Italy; ^2^Section of Neurophysiopathology, Department of Clinical and Experimental Medicine, University of Pisa, Pisa, Italy

**Keywords:** Huntington's disease, neurodegenerative diseases, cerebellum, tDCS, rTMS, non-invasive brain stimulation

## Abstract

**Introduction:** In recent years, a growing body of literature has investigated the use of non-invasive brain stimulation (NIBS) techniques as a putative treatment in Huntington's Disease (HD). Our aim was to evaluate the effects of cerebellar transcranial Direct Current Simulation (ctDCS) on the motor outcome in patients affected by HD, encompassing at the same time the current knowledge about the effects of NIBS both on motor and non-motor dysfunctions in HD.

**Materials and Methods:** Four patients (two females) were enrolled and underwent ctDCS (both anodal or sham, elapsed by at least 3 months: 2.0 mA, 20 min per day, 5 days a week). Clinical scores were assessed by using the Unified Huntington's Disease Rating Scale – part I (UHDRS-I), immediately before ctDCS (T_0_), at the end of the 5-days treatment (T_1_) and 4 weeks later (T_2_).

**Results:** Anodal ctDCS improved motor scores compared to baseline (*p* = 0.0046), whereas sham stimulation left them unchanged (*p* = 0.33, Friedman test). In particular, following anodal ctDCS, UHDRS-I score significantly improved, especially regarding the subitem “dystonia,” both at T_1_ and T_2_ compared to sham condition (*p* < 0.05; Wilcoxon matched-pairs signed test).

**Conclusions:** ctDCS improved motor scores in HD, with effects lasting for about 4 weeks after tDCS completion. This is the first study discussing the putative role of cerebellar non-invasive simulation for the treatment of HD.

## Introduction

Huntington's disease (HD) is a neurodegenerative, progressive and fatal disorder, clinically characterized by cognitive impairment, behavioral dysfunctions and hyperkinetic movements. Its prevalence is higher than previously thought, geographically variable and increasing worldwide (1, 2). The disease is caused by an expanded CAG trinucleotide repeat (of variable length) in HTT, the gene encoding the protein *huntingtin*; it is inherited in an autosomal dominant manner, with age-dependent penetrance, where longer CAG repeats predicting earlier onset (3). HD belongs to the so-called “poly-glutamine disorders,” a heterogeneous group of diseases caused by an abnormal expansion of a glutamine encoding CAG repeat in the affected genes. To date, disease modifying therapies are not yet available and the pharmacological treatment is only symptomatic. Also non-invasive brain stimulation techniques (NIBS), as repetitive Transcranial Magnetic Stimulation (rTMS) and transcranial Direct Current Stimulation (tDCS), have reported conflicting results in the treatment of severe choreic and dystonic movements, failing to provide a significant, possibly long-lasting, clinical improvement in HD patients (4–8).

The multisystem character of HD is emphasized by a distribution pattern of neurodegeneration which includes not only the striatum, but also the cerebral neo-and allocortex, thalamus, pallidum, brainstem and the cerebellum, thus sharing more similarities with polyglutamine spinocerebellar ataxias than previously described (9). A possible clinical and pathogenetic overlap has been recently supported also by neurophysiological findings (10, 11).

Recently, a growing attention has been focused on the cerebellar involvement in the pathogenesis of HD (12–14). Clinically, several symptoms of HD could be attributed to cerebellar damage, comprising dysarthria, ataxia, gait disturbances and abnormal oculomotor function. Moreover, animal studies have strengthened the possibility of a key cerebellar involvement in HD models (15–17). Magnetic resonance imaging (MRI) studies have further corroborated these data in humans, showing that aberrant cerebellar diffusion and smaller cerebellar volumes are associated both with a worst motor performance and increased psychiatric symptoms at early stages (12).

To date, only few papers have investigated the role of non-invasive brain stimulation in HD and no studies targeted the cerebellum. In this pilot trial, our purpose was to assess the possibility to improve motor symptoms with cerebellar transcranial Direct Current Stimulation (ctDCS) in clinical manifest HD, encompassing the current literature about the use of cerebellar NIBS for the treatment of HD. Patients underwent anodal and sham (placebo) ctDCS; although there are different factors driving the direction of ctDCS after-effects, as the electrode size and the montage, anodal stimulation is known to exert an overall excitatory effect on cerebellar functions, both motor and non-motor (18–21).

## Materials and Methods

### Patients

Four HD patients were enrolled in a timeline ranging from April 2016 to November 2018. They had undergone genetic testing, which was diagnostic in all (CAG number ⩾ 40). The mean duration of symptoms was about 2 year (23.3 ± 7.8 months). Inclusion criteria for the early manifest HD patients were a CAG repeat ⩾ 40, with a UHDRS motor score ⩾ 5, and a total functional capacity (TFC) score ⩾ 7. Demographic and clinical data are summarized in Table 1. The pharmacological therapy remained the same during the whole experimental protocol.

**Table 1:** Demographic and clinical features of HD patients.

**Patient**	**1**	**2**	**3**	**4**
Age	45	50	43	48
Sex	F	F	M	M
MMSE	26/30	27/30	27/30	28/30
CAG-length	44	41	41	45
UHDRS-I (motor score)	24	22	16	23
Onset of motor symptoms	4.0 years	3.5 years	3.5 years	4.5 years
Pharmacological therapy	Tetrabenazine 75 mg/day	Tetrabenazine 37.5 mg/day	Tetrabenazine 37.5 mg/day	Tetrabenazine 50.0 mg/day
Timeline of intervention	Anodal/sham	Sham/anodal	Anodal/sham	Sham/anodal

*MMSE, Mini-Mental State Examination; UHDRS-I, Unified Huntington's Disease Rating Scale part 1; CAG-length, mutational load expressed as the number of CAG repetitions in the target gene*.

The patients were enrolled and the experimental procedures performed at the Section of Neurophysiopathology, University of Pisa.

### Experimental Protocol

In a crossover, double-blind, sham-controlled design, each patient underwent sham and anodal ctDCS. All patients carried out the two experimental conditions, held at least 3 months apart to avoid carry-over effects. Each session, either anodal or sham, lasted 5 days a week (Monday to Friday, 30 min a day).

Clinical scores were assessed at baseline (T_0_), immediately at the end of the stimulation week (T_1_), 4 weeks (T_2_) later.

Patients were enrolled by a physician with expertise in movement disorder medicine (F.S.); clinical scores were administered by a neurologist (T.B.), blinded to the ctDCS condition. A third neurologist with expertise in movement disorders (A.P.) served as blinded video-rater and confirmed the motor outcome.

Informed consent was obtained from all individual participants included in the study. The study was approved by the local ethical committee at the University of Pisa (formally named “Comitato Etico di Area Vasta Nord Ovest della Toscana”), in accordance with the tenets of Helsinki.

### Transcranial Direct Current Stimulation

Cerebellar transcutaneous direct current stimulation (ctDCS) was applied using a battery-driven constant current stimulator (HDCStim, Newronika®, Italy) and a pair of electrode in two saline-soaked synthetic sponges with a surface area of 35 cm^2^. Direct current was transcranially applied for 20 min with an intensity of 2.0 mA and constant current flow was measured by an ampere meter (current density ~0.08 mA/cm^2^). At the offset of tDCS, the current was decreased in a ramp-like manner, a method shown to achieve a high level of blinding between sessions (18). For anodal ctDCS, the anode was applied on the median line, 2 cm below the inion, with lateral borders about 1 cm medially to the mastoid apophysis, and the cathode over the right shoulder (19, 21–25). For sham ctDCS, the current was turned on for 5 s and then turned off in a ramp-shaped fashion, thus inducing skin sensations similar to those produced by real ctDCS.

We stimulated the cerebellum bilaterally, as previous studies have shown that varying the position of the active electrode with ~1 cm induced negligible changes in the electrical field distribution (26).

Patients were blinded to the tsDCS protocol and did not discriminate between anodal and sham condition. In order to report possible adverse effects, the questionnaire developed by Brunoni et al. was administered to each patient (27).

### Clinical Outcome and Statistical Analysis

The clinical outcome was assessed by evaluating the Unified Huntington Disease Rating Scale-part I (UHDRS-I) score, particularly its “dystonia” (cumulative values for trunk and extremities) and “chorea” (face, mouth, trunk and extremities) subscores, at the different time points (T_0_, T_1_, and T_2_) (28). ctDCS-induced changes in UHDRS-I score and related sub-items were assessed with a Friedman test (non-parametric analysis on paired data) with the main factor “time” (three levels: *T*_0_, *T*_1_, and *T*_2_). In order to disclose significant changes at each time point between anodal and sham ctDCS (*T*_0_, *T*_1_, *T*_2_,), a Wilcoxon matched-pairs signed test was then applied. Statistical significance was set at *P* < 0.05. The data were analyzed using SPSS v. 21.0 for Windows (SPSS Inc.). Raw data are shown as mean values ± 1 standard error (S.E.).

## Results

No patient reported adverse effect during stimulation or the follow-up period.

**Figure 1:**
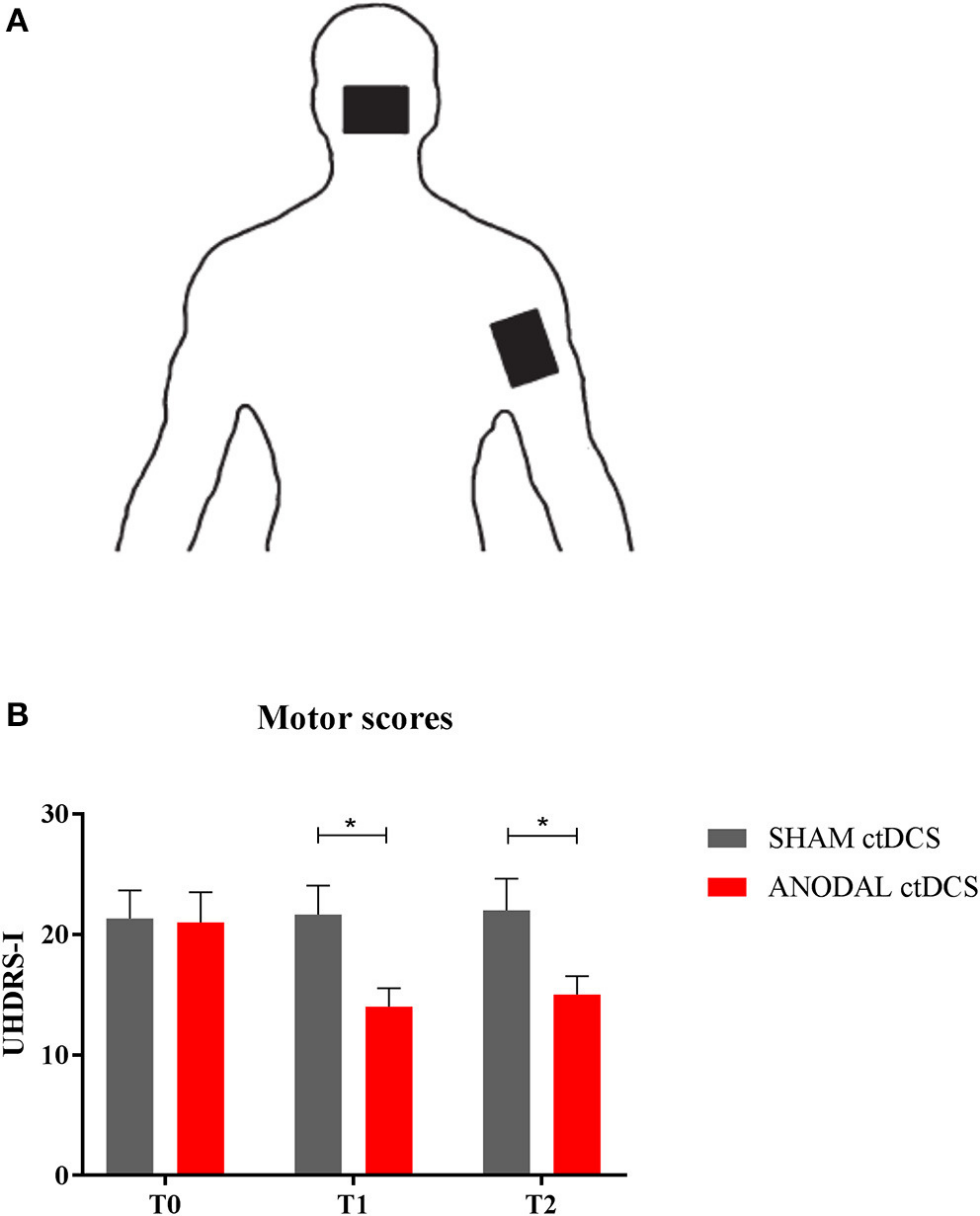
**(A)** ctDCS montage. The active electrode was placed over the cerebellar area, while the return electrode was positioned over the right shoulder. **(B)** Motor score changes. The histogram shows UHDRS-I values at different time intervals (T_0_, T_1_, and T_2_), following either sham (gray columns) or anodal ctDCS (red columns). Note that real (anodal) stimulation significantly reduced motor impairment, both at T_1_ and T_2_. Data are shown as mean values ± 1 S.E. (*p < 0.05).

Figures 1A,B shows changes in UHDRS motor score (UHDRS-I) following either sham (gray columns) or anodal ctDCS (red columns).

Overall, compared to baseline (T_0_), anodal ctDCS improved motor scores (UHDRS-I: T_0_ = 22.0 ± 1.9; T_1_ = 14.5 ± 1.2; T_2_ = 15.5 ± 1.1: *p* = 0.0046), whereas sham stimulation left them unchanged (T_0_ = 21.7 ± 1.7; T_1_ = 22.1 ± 1.7; T_2_ = 22.5 ± 2.6: *p* = 0.33, Friedman test). In particular, following anodal polarization, UHDRS-I score significantly improved both at T_1_ and T_2_ compared to sham tDCS (*p* = 0.46 and *p* = 0.48, respectively; Wilcoxon matched-pairs signed test).

**Figure 2:**
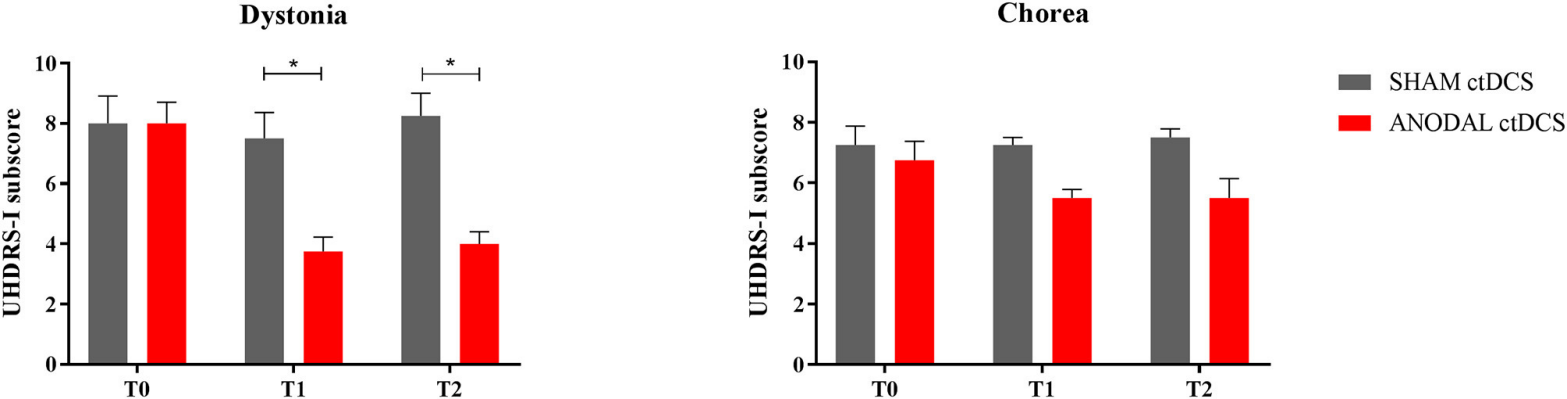
Motor scores changes in the subitems “dystonia” (left) and “chorea” (right). The histogram shows UHDRS-I subscores at different time intervals (T_0_, T_1_, and T_2_), following either sham (gray columns) or anodal ctDCS (red columns). Note that real (anodal) stimulation significantly reduced dystonia, with a smaller effect on choreic movements. Data are shown as mean values ± 1 S.E. (*p < 0.05).

As regards UHDRS-1 subscores, anodal ctDCS significantly improved dystonia over time (T_0_ = 8.0 ± 0.7; T_1_ = 3.7 ± 0.5; T_2_ = 4.5 ± 0.4: *p* = 0.037), whereas sham stimulation had no significant effects (T_0_ = 8.0 ± 0.9; T_1_ = 7.5 ± 0.9; T_2_ = 8.3 ± 0.7: *p* = 0.29, Friedman test; Figure 2). In particular, following anodal polarization, dystonia was reduced both at T_1_ and T_2_ compared to sham tDCS (*p* = 0.46 and *p* = 0.48, respectively; Wilcoxon matched-pairs signed test).

Conversely, both active and sham ctDCS left chorea scores unchanged over time, whereas a trend to improve was shown following the anodal stimulation (anodal ctDCS: T_0_ = 6.7 ± 0.6; T_1_ = 5.5 ± 0.3; T_2_ = 5.5 ± 0.6: p = 0.074; sham ctDCS: T_0_ = 7.3 ± 0.6; T_1_ = 7.3 ± 0.3; T_2_ = 7.5 ± 0.3: *p* = 0.8).

## Discussion and a Reappraisal of the Current Literature

Cerebellar direct current polarization improved motor scores in HD patients, especially dystonia, with the effects lasting for 1 month after the end of stimulation. To the best of our knowledge, this is the first report showing a significant effect of cerebellar stimulation on motor scores in HD. Only few papers have evaluated NIBS techniques for the treatment of both motor and non-motor symptoms in HD patients (Table 2). Among these, Brusa and colleagues found an improvement of choreic movements by 1 Hz rTMS, when applied over the supplementary motor area (SMA) (6); conversely, Shukla and co-workers showed that bilateral low-frequency rTMS over the SMA did not reduce choreiform movements in severe HD (5). A third, single-case study has recently proposed the use of “deep” TMS (dTMS, at 1 Hz and 120% of the motor threshold) for the treatment of depression and anxiety in HD (31). To support beneficial effects induced by TMS on HD patients, some Authors have found that Transcranial magnetic stimulation attenuates cell loss, oxidative and nitrosative damage in the striatum of a murine HD model (29). More recently, it has been demonstrated that anodal tDCS enhances working memory in HD, when applied over the left dorsolateral prefrontal cortex (DLPFC), and this improvement is greatest in patients with more severe motor symptoms, thus suggesting that motor scores may help identify patients who are most likely to benefit from tDCS (4).

**Table 2:** Non Invasive Brain Stimulation (NIBS) for the treatment of HD: current literature.

**Author, date**	**Sample**	**Methods**	**Follow-up period**	**Primary outcome**	**Main results**
**tDCS**					
Eddy et al. (4)	20	Sham vs. 1.5 mA anodal tDCS on the left DLPC	Immediately post-rTMS	Working memory	Anodal tDCS improves working memory, especially in patients with more severe motor symptoms.
**rTMS**					
Brusa et al. (6)	8	1 Hz rTMS on SMA	Immediately post-rTMS (30′)	Choreic movements	1 Hz rTMS improves choreic movements.
Túnez et al. (29)	n.a.	Murine model: high-frequency rTMS (60 Hz), applied for 4 h a day	Immediately post-rTMS	Oxidative stress markers	rTMS attenuates cell loss, oxidative and nitrosative damage in the striatum.
Shukla et al. (5)	2	Seven consecutive sessions of bilateral 1 Hz rTMS on SMA (900 pulses)		Choreic movements	No effects on choreic movements in severe HD.
Davies et al. (30)	Single case	“Deep” rTMS on SMA (1 Hz at 120% RMT; 1600 pulse for 49 daily session)	8 months	Depression and Anxiety	Improvement of depression and anxiety scores following the real stimulation.

*tDCS, transcranial Direct Current Stimulation; SMA, Supplementary Motor Area; RMT, Resting Motor Threshold; DLPC, dorsolateral prefrontal cortex; HD, Huntington's Disease; n.a., not applicable as the paper refers to a murine model*.

The finding that ctDCS significantly improved specific subscores than others fits with recent data showing a key cerebellar involvement in the pathophysiology of dystonia (32–36), especially in inherited disorders (37–41). In particular, the cerebellum is likely engaged in a hyper-direct, short-delay cerebello-talamo-striatal pathway, projecting from the dentate nucleus to the striatum and the external segment of the globus pallidus (GPe), via the intralaminar nuclei of the thalamus (42, 43). Under pathological conditions, this pathway can bypass the cerebello-thalamo-cortical stream and drive abnormal activity within the basal ganglia, thus inducing dystonic movements (43).

Recently, an increasing body of literature has specifically investigated the relationship between HD pathogenesis and the cerebellum.

Both autoptic and MRI studies have suggested that cerebellar abnormalities are present in HD at early stages, mainly involving the gray matter of the deep cerebellar nuclei, with a relative preservation of the cerebellar cortex (9, 44–47). The cerebellar involvement is particularly marked, both from a clinical and a neuropathological perspective, especially in the Juvenile Huntington's Disease (JHD), a rare HD variant arising before 20 years age and characterized by a variable presentation, including myoclonus, seizures, Parkinsonism, and cognitive decline (48–51).

Nonetheless, how these abnormalities play a contributing role in the pathophysiology of the disease is still a matter of debate. From a neuropathological point of view, HD is characterized by a bilateral and symmetrical neuronal loss in the neostriatum, mainly caused by the extensive demise of GABA-ergic medium spiny stellate projections neurons (52–57). The lack of any inhibitory cerebellar effect in patients with dystonia may contribute to the loss of M1 inhibition and the development of incorrect motor programs and maladaptive behaviors (58, 59). Anodal ctDCS may ultimately interfere with cerebello-thalamo-cortical loops, possibly restoring the physiological inhibition exerted by cerebellar nuclei on cortical processing.

Moreover, as described above, the cerebellum itself directly interferes with striatal networks through a disynaptic pathway leading to the intralaminar nuclei of the thalamus and to the dorsolateral putamen (42).

## Limitations

Our study has some limitations. First, the small sample, given that our study has been designed as a pilot research; nonetheless, other studies have enrolled a similar number of patients, probably due to the fact that HD is a quite rare disease. Second, other clinical measures (e.g., quality of life and depression) were not assessed and included as secondary outcomes. Third, a longer observation period should be used in further studies, in order to assess how long the beneficial effects of cerebellar tDCS persist. As a pilot trial, further studies are needed.

## Conclusions

This is the first pilot trial about the use of cerebellar tDCS for the treatment of motor symptoms in HD. Further and large-scale studies are needed, possibly including non-motor features as secondary outcomes. Moreover, the impact of a combined cerebello-cortical stimulation may be of interest, in order to improve therapeutic effects on motor scores.

## Data Availability Statement

The raw data supporting the conclusions of this article will be made available by the authors, without undue reservation.

## Ethics Statement

The studies involving human participants were reviewed and approved by Università di Pisa. The patients/participants provided their written informed consent to participate in this study.

## Author Contributions

TB and FS: concept and design. TB and DB: acquisition of data. TB, DB, and RF: acquisition and interpretation of data. TB, RF, AP, and FS: drafting of the manuscript. AP: critical revision of the manuscript for important intellectual content. All authors contributed to the article and approved the submitted version.

## Conflict of Interest

The authors declare that the research was conducted in the absence of any commercial or financial relationships that could be construed as a potential conflict of interest.
